# Present and Perspectives of Photoactive Porous Composites Based on Semiconductor Nanocrystals and Metal-Organic Frameworks

**DOI:** 10.3390/molecules26185620

**Published:** 2021-09-16

**Authors:** Alejandro Cortés-Villena, Raquel E. Galian

**Affiliations:** Institute of Molecular Science, University of Valencia, c/ Cat. José Beltrán 2, 46980 Paterna, Valencia, Spain; alejandro.cortes@uv.es

**Keywords:** photoactive materials, MOFs, perovskites, MHP@MOF composites, photoluminescence, synergism

## Abstract

This review focuses on the recent developments in synthesis, properties, and applications of a relatively new family of photoactive porous composites, integrated by metal halide perovskite (MHP) nanocrystals and metal-organic frameworks (MOFs). The synergy between the two systems has led to materials (MHP@MOF composites) with new functionalities along with improved properties and phase stability, thus broadening their applications in multiple areas of research such as sensing, light-harvesting solar cells, light-emitting device technology, encryption, and photocatalysis. The state of the art, recent progress, and most promising routes for future research on these photoactive porous composites are presented in the end.

## 1. Introduction

Photoactive semiconductor nanocrystals, light-responsive materials in the nanoscale regime, exhibit unique optical properties in the ultraviolet-visible or near-infrared region according to their nature, composition, shape, and size [1]. We have witnessed great success in the last two decades in the field of metal chalcogenide quantum dots (QDs). Over the last few years, metal halide perovskite (MHP) nanocrystals have emerged as an exciting new class of semiconductor materials with outstanding electronic and optical properties, easy synthesis, and high photoluminescence quantum yield. Despite great progress in the field, the long-term phase stability of perovskite-based materials remains a big challenge and compromises their practical application [2,3]. To overcome this problem, device encapsulations, such as glass-glass, polymer, and direct thin-film encapsulation, have been proposed to improve the stability of perovskite solar cells [4].

Despite the potentials of MHP nanocrystals, the problems associated with their environmental instability when exposed to different stress conditions, such as moisture, oxygen, UV-light irradiation, and heat, have yet to be resolved [5]. Significant efforts have been devoted to improving the water stability of MHP nanocrystals by various strategies such as encapsulation in a macrocyclic host [6], incorporation into oligomer matrix [7], and post-synthetic surface treatment [8]. However, the organic materials often used as host matrix have poor thermal resistance, which could limit their applications. In contrast, inorganic host matrices such as SiO_2_ [9] and zeolites [10] exhibit better thermal resistant properties, but the remarkable features of MHPs are sometime difficult to fully preserve. This crucial drawback might somehow be addressed by the encapsulation of MHP nanocrystals into protective porous materials, such as metal-organic frameworks (MOFs) [11], or inorganic shell coatings to obtain core/shell structures [12].

Metal-organic frameworks (MOFs) constitute a class of porous materials composed of multidentate ligand connectors and metal ions or metal clusters, with adjustable pore size, specific surface area, large pore channels, and excellent physical and chemical properties. These features have made them interesting in catalysis, energy, membrane separation, and biology [13]. Therefore, the structural and chemical diversity of MOFs makes them a very attractive platform for integrating semiconductor nanomaterials into their pores, resulting in novel photoactive porous composites. The MOF matrix could help to prevent the agglomeration, reduce the photoluminescence quenching, and improve the extrinsic and intrinsic stability of the incorporated nanoparticles.

This review aims to provide an overview of the synthesis methodologies used to successfully prepare photoactive porous composites. The incorporation of MHP nanocrystals into MOF structures improves their emissive properties and stability. An update of the recent examples reported in recent years with applications in LED technology, photovoltaics, sensing, encryption, and photocatalysis will be summarized. Finally, a perspective on future research directions of photoactive porous composites (MHP@MOF) will be commented on.

### 1.1. Photoactive Semiconductor Nanocrystals

Luminescent semiconductor nanocrystals, usually known as quantum dots (QDs), have demonstrated outstanding progress since their discovery in the early 1980s [14]. Generally, they possess diameters smaller than the exciton Bohr radius (below 10 nm), which endows them with remarkable optical and electronic properties due to quantum confinement effects, and present discrete electronic energy levels that can be precisely controlled by the nanocrystal size and shape [15,16]. Therefore, the fact that they have easily tuneable and size-dependent photophysical properties makes quantum dots of great interest in various fields of application such as flexible transistors [17], solar cells [18], light-emitting diodes (LEDs) [19], lasers [20], quantum computing [21], photodetectors [22], catalysis [23], cell biology research, imaging, and diagnostics [24]. 

The quantum dot composition can be varied and formed predominantly with elements of groups 12–16 (CdS, CdSe, CdTe, ZnS, ZnSe), 13–15 (GaN, InP, InAs), or 14–16 (PbS, PbSe). Some of the attractive properties of QDs include the broad absorption, narrow and mostly symmetrical emission bands, large molar absorption coefficients (10^5^–10^6^ M^−1^·cm^−1^), low photobleaching, long lifetimes (from five to hundreds of nanoseconds), and good photoluminescence quantum yields [25]. QDs can be classified into three types: (i) a core QD structure consisting of a single material such as metal chalcogenide, a typical example of which is CdSe QDs; (ii) core/shell QD structure composed of an inorganic core encapsulated inside another semiconducting nanocrystal with a higher bandgap, such as CdSe/ZnS QDs; and (iii) alloy QD structure, which consists of a homogeneous mixture of semiconducting nanocrystals such as CdSe_1-x_Te_x_ [26]. One of the main issues with QDs is their non-tolerant nature to surface defects caused by dangling bonds of ions at the surface that reduce their photoluminescence properties [27]. The most promising strategy to improve the QDs’ photoluminescence is surface passivation with a semiconductor material shell with a suitable bandgap, which suppress the non-radiative pathways and enhances their chemical stability [28,29]. Much progress has been made over the past 20 years on synthesis approaches (bottom-up and top-down) to yield uniform, high-quality colloidal QDs. While bottom-up strategies led to a uniform and better-controlled nanoparticle size, they are limited by their scalability [30,31].

More recently, perovskite materials have emerged as a very promising light harvester in solar cells owing to their outstanding optoelectronic properties, i.e., a high absorption coefficient, low recombination losses, high defect tolerance, low-cost processing, long charge diffusion lengths, and easy bandgap tunability [32]. The first example using hybrid CH_3_NH_3_PbI_3_ perovskite in a solar cell was reported by Miyasaka’s group in 2009, with a power conversion efficiency (PCE) value of 3.8% [33]. Currently, a PCE of up to 25.6% has been achieved, which is comparable to single-crystal silicon solar cells, making MHP the fastest growing material in the field of photovoltaics [34].

MHP nanocrystals (MHP NCs) have become very attractive as a new class of fluorophore with a large absorption coefficient, symmetrical and narrow emission spectrum, and high photoluminescent quantum yield (Φ_PL_) [35]. Beyond their solar cell application [36], they have been extensively exploited in diverse fields such as light-emitting device (LED) technology [37], displays [38], lasers [39], catalysis [40], etc. [41].

**Figure 1:**
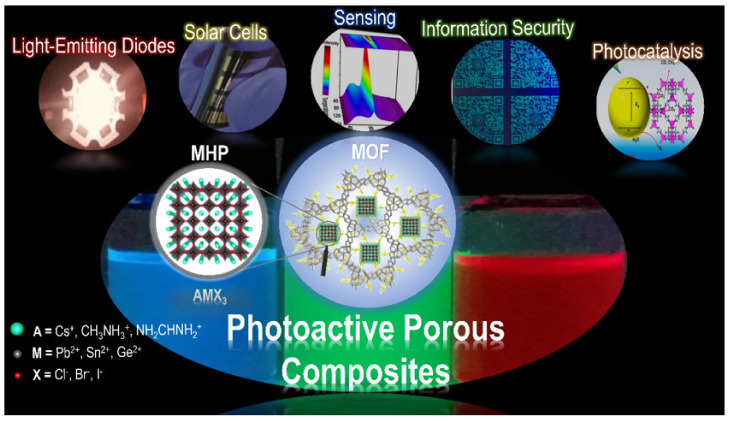
Schematic illustration of the photoactive porous composites (MHP@MOF), based on metal halide perovskite (MHP) and porous metal-organic framework (MOF), and their main applications.

MHP materials present a general crystalline structure with the formula AMX_3_ that resembles that of the original oxide perovskite CaTiO_3_ discovered in 1839 [42]. In the crystalline structure (Figure 1), “A” stands for monovalent cations (i.e., cesium, rubidium, methylammonium, or formamidinium), “M” for divalent cations (i.e., lead, tin, germanium), and “X” for monovalent anions, generally halides (i.e., chlorine, bromide, iodide, and/or a mixture of them). Depending on the nature of “A” cations, they can be classified as all-inorganic or organic–inorganic hybrid metal halide perovskites [43]. The AMX_3_ structure involves an infinitely extended 3D array made up of corner-sharing MX_6_ octahedra, in which the A-site cations occupy the cuboctahedra (12-fold) voids created by the MX_6_ octahedral (6-fold) framework [44]. Perovskites can be categorized by the size of the material as (i) bulk 3D perovskites; (ii) low-dimensional materials, 2D as nanoplatelets and nanosheets, and 1D as nanowires and nanorods; and (iii) 0D materials as nanoparticles. A special feature of MHPs is that they can be assorted according to the dimensionality of the inorganic framework: as the size of the A cation increases, the 3D array will change to a 2D framework (MX_6_ octahedra are connected in layered sheets at their corners), 1D framework (MX_6_ octahedra are connected at the corners, edges, or faces), or 0D framework (isolated MX_6_ octahedra) [45]. Similar to metal chalcogenide QDs, the bandgap of MHPs, and therefore the optical and electronic properties, can be precisely tuned over the entire visible spectral range by the perovskite composition, size, and shape and according to the dimensionality of the inorganic framework [45,46]. The intrinsic stability of perovskite structures can be predicted by the Goldschmidt tolerance factor (t) based on the radii of their cations and anions [47]. Thanks to the mostly held ionic bonding, MHPs can be synthesized even at low reaction temperatures (i.e., room temperature) in the form of colloidal nanocrystals, single crystals, and thin films [48]. However, the high-temperature synthesis methodologies, prepared in the presence of organic ligands, have the advantage of producing high-quality MHP nanocrystals with uniform size and shape [35]. These capping agents play a key role in nanoparticle growth control and crystallization, as well as preventing agglomeration and providing processability and functionalization, therefore determining the nanocrystals’ performance and their final application [49]. Since the first report on the synthesis of colloidal MHP nanocrystals in 2014 through a reverse microemulsion strategy [50], there have been several synthesis approaches developed in solution involving bottom-up methods. The reverse microemulsion strategy is a nontemplate method consisting of mixing the perovskite precursors (CH_3_NH_3_Br and PbBr_2_ dissolved in dimethylformamide) in octadecene (ODE) and oleic acid (OA) at 80 °C, in the presence of octylammonium bromide as an organic ligand, followed by the addition of acetone as a polar solvent to induce the MHP crystallization [50]. Soon after, the ligand-assisted reprecipitation (LARP) method [51,52] was reported, where a precursor solution of perovskite sources (PbX_2_ and CsX) and ligands (alkyl acids and alkyl amines) is first dissolved in a “good” solvent and then added to a “bad” solvent, under stirring at room temperature, to induce crystallization of the perovskite material. In the hot-injection method, a pre-synthesized cesium oleate solution is swiftly injected into an octadecene solution of PbX_2_, oleic acid, and oleylamine (OAm) at a high temperature under inert conditions, followed by cooling down the reaction flask in an ice bath [53].

One of the most striking features of MHP nanocrystals is their defect tolerance nature [35], i.e., their optoelectronic properties are mostly unaffected by the defects, which tend to be either localized within the valence and conduction bands or to be essentially inert, resulting in MHP nanocrystals with higher Φ_PL_ than traditional metal chalcogenide QDs. As a consequence of this benefit, MHPs exhibit bright photoluminescence without the need for surface passivation through shell growth to produce high-performance NCs [54]. Nevertheless, the lead toxicity limits the development of safe perovskite-based technologies and bioapplications. This shortcoming has stimulated extensive research in various directions to replace lead with other cations such as germanium and tin as well as the preparation of double perovskites as a green alternative to lead halide counterparts [55,56].

### 1.2. Metal-Organic Frameworks (MOFs)

Metal-organic frameworks, a term introduced in 1995, constitute a family of metal-organic materials (MOM) which are built by inorganic nodes (metal ions or clusters) known as secondary building units and organic linkers which assemble into 3D network-type structures with a large surface area and considerable pore volume [57]. Generally, most of the reported MOFs show microporous characters (<2 nm), and only a small fraction of MOFs with mesoporous structures has been reported (2–50 nm) [58]. Transition metals, actinides, alkaline earth metals, and mixed metals are often used as inorganic nodes, whereas carboxylates, sulphates, phosphonates, azoles, and heterocyclic compounds are commonly employed as organic linkers [59]. Strong coordination bonds should be rationally designed for MOFs to be stable enough and avoid side reactions. In light of hard/soft acid-base (HSAB) theory, there is a preference for hard acids to bind with hard bases, providing additional stability in terms of bond dissociation energy [60]. Hence, high-valent metal ions (e.g., Zr^4+^, Al^3+^, Cr^3+^, Ti^4+^, Fe^3+^) usually possess high charge densities and coordination numbers, thus benefitting the formation of strong coordination bonds and rigid structures when binding to hard bases [61]. From the point of view of supramolecular chemistry, when compared with conventionally used microporous inorganic materials such as zeolites, MOFs have the potential for a more flexible rational design, tuneable structure, large surface area (up to 10,000 m^2^/g), variable pore diameters (from micro- to mesoporous), and tailorable functionalities [62], which endow MOFs with a variety of tuneable properties such as charge, polarity, chirality, redox potential, photoactivity, hydrophobicity/hydrophilicity, aromatic/lipophilic character, stereochemistry, and so on [63]. Due to all these features, MOF materials have widely been used in a range of potential applications concerning catalysis [64,65,66,67], gas adsorption, storage and release [68], molecular separation [69,70], sensing [71], lighting [72], therapeutics, drug carriers, imaging, and biosensors in biomedicine [73].

The first studies on MOFs began in the late 1980s [74], and from there, MOFs with different topologies and functionalities have been prepared [75,76,77,78,79]. The modulation of the length of organic linkers made it possible to precisely control the pore size, providing them with structures with large porosities and surface area [80].

There are many strategies for synthesizing MOFs with different features, i.e., solvothermal, microwave, slow evaporation, mechanochemical, sonochemical, electrochemical, etc., that will affect the final properties of MOFs [81].

The solvothermal method is the most widely used for synthesizing MOFs with various morphologies, offering precise control over morphology, crystallinity, and size [82]. It consists of dissolving a metal salt and organic ligands in an organic solvent. Due to high reaction temperatures, high-chemical yield materials can be obtained. Nevertheless, after synthesizing the corresponding MOF by this method, it is necessary to remove solvent molecules from the pores. This process can be carried out through vacuum drying or washing with a solvent such as ethanol or methanol. The microwave-assisted method is known to be a very fast and simple method to generate MOFs, owing to the microwave power, to shorten reaction times and produce highly crystalline and porous textures with shape and particle size control [83]. When using the slow-evaporation method [84], MOFs are produced by slow solvent evaporation. Although this strategy is advantageous from the point of view that no external energy is applied, it is time-consuming. The mechanochemical strategy is a more sustainable approach in which MOFs are synthesized by mechanical agitation between precursors in the absence of toxic solvents [85]. The sonochemical method produces MOFs using high-frequency ultrasonic waves with decreased crystallization time compared to the conventional solvothermal method [86]. These ultrasonic waves cause the formation and collapse of small bubbles, creating short-lived local hot spots at high temperatures and pressures, resulting in uniform nucleation. The electrochemical strategy generates metal ions from the electrode when an appropriate voltage/current is applied, and then these metal ions react with the organic linker in solution to form the corresponding MOF close to the electrode surface [87].

Based on the stability and dynamic nature of the framework, MOF materials can also be classified according to Kitagawa’s categories of porous coordination polymers [88]. In the case of 1st generation, MOFs irreversibly lose their crystallinity, undergo a phase change, or alter their morphology upon removal of guest molecules from their framework, in contrast to zeolitic materials. However, in the case of the 2nd generation, MOFs possess relatively stable frameworks and do not change after the removal of guest molecules. In the 3rd generation, MOFs exhibit dynamic and flexible properties, which change their frameworks, responding to guest exchange or external stimuli such as pressure, light, etc. [88]. Finally, 4th generation MOFs are correlated to the recently developed post-synthesis modifications which can maintain underlying topology and structural integrity towards several post-modifications [63].

### 1.3. Photoactive Porous Composites Based on Semiconductor Nanocrystals and Metal-Organic Frameworks

There are a large number of MOF families well studied in the literature such as Zr-oxide nodes (e.g., UiO-66), Cu-Cu paddlewheels (e.g., HKUST-1), ZIF-like, Zn-oxide nodes, IRMOF-like, and MOF-74/CPO-27-like materials [89]. Thanks to research efforts on the composition and structural diversity of MOFs, they can be designed and synthesized with properties and functionalities suitable for the integration of certain guests into their porous cavities as nanoparticles, improving the stability of the incorporated material [59].

A variety of MOF composites, resulting from their combination with other materials, including polymers [90], some carbon-based nanomaterials such as carbon nanotubes (CNTs) [91] and graphene [92], polyoxometalates (POMs) [92], biomolecules [93], metal nanoparticles [94], quantum dots (QDs) [95], and more recently MHP nanocrystals, have been studied [96].

The preparation of QD@MOF composites using metal chalcogenide QDs as a photoactive semiconductor nanocrystal has been extensively explored. Several examples integrate QDs into an MOF matrix to improve their stability and reduce photoluminescence quenching, with multiple applications in sensing, light-harvesting, and photocatalysis [95,97,98,99,100].

Nevertheless, new functional photoactive porous composites (MHP@MOF) are currently under investigation, which combine the fascinating optical properties of MHPs and the encapsulation ability of MOFs, as a promising strategy to improve perovskite stability [80]. In the following sections, we will describe the synthesis methodology and the state of the art of MHP@MOF composites in light-emitting diode technology, solar cells, sensing, information security, and photocatalysis (Figure 1). The optical properties of photoactive porous composites will be discussed, with particular emphasis on the synergistic effects over their stability and photoluminescent features.

Photoactive porous composites based on MHP have demonstrated very promising applications as light-harvesters in the photovoltaic field and as good emitters for photoluminescence-based technologies. The optoelectronic properties of MHP have been improved in the presence of MOF, reaching power conversion efficiency values of ca. 21% and good stability over time under ambient atmosphere (up to 30 days). MOFs play a key role in the nucleation and morphology of MHPs, improving the quality of the perovskite film. However, some issues need to be overcome such as long-term phase stability, reduction of voids between grains, and MHP crystallinity. Furthermore, the defect passivation of the perovskites using MHP/MOF heterojunction could be improved, raising the resistance of the device towards humidity, heat, and light-irradiation. It is noteworthy that most of the emissive MHP@MOF composites reported up to now are based on lead bromide perovskite nanocrystals, APbBr_3_ (A = CH_3_NH_3_^+^ or Cs^+^), which present a good photoluminescence response in the green region (Φ_PL_ = 39–72%). They have been successfully combined with blue and red organic emitters to build white LED devices with good luminous efficiency. Although the resulting MHP@MOF composites described in Table 1 are very promising for LED technology, further fundamental studies and optimization of the synthesis approaches are required to improve the composite photophysical properties and achieve superior intrinsic and extrinsic MHP stability.

Dual emissive MHP@MOF composites have been proposed as good candidates for information security applications. Interestingly, the enhanced photoluminescence of MHP in the composite can be achieved, promoted by the energy transfer from the organic linker in the MOF towards MHP nanocrystals. Dual emission has been also exploited for selective sensing of metal ions in aqueous media and as an optical temperature sensor. 

Regarding the photocatalytic application of MHP@MOF composites, a few examples have been reported such as the reduction of CO_2_ and the degradation of environmental pollutants. The photocatalysis performance of MHP has been remarkably enhanced in the MOF matrix, owing to the porous structure with a large surface area and number of active sites. 

## 2. Synthesis Methodology of Photoactive Porous Composites

**Figure 2:**
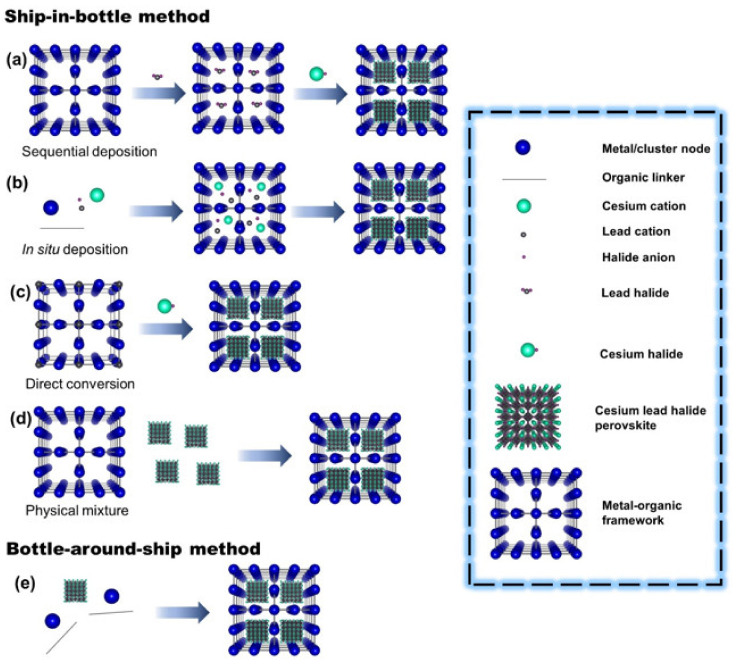
Synthesis methodologies for preparing MHP@MOF composites: (**a**–**d**) ship-in-bottle methods including sequential deposition, in situ deposition, direct conversion, and physical mixture, and (**e**) bottle-around-ship method.

Different strategies for synthesizing MHP@MOF composites have been established since there are several ways to incorporate guest species into the MOF host matrix. In particular, perovskite nanocrystals can be directly synthesized and integrated into MOF pores by roughly ship-in-bottle and bottle-around-ship methodologies [133] as shown in Figure 2. Regardless of the strategy used, MHP@MOFs offer higher stability to the MHP against ambient and working conditions. Table 1 summarizes the MHP@MOF composites reported in the literature assorted by their application. Moreover, the synthetic methodology as well as the optical structural features are noted.

### 2.1. Ship-in-Bottle Method

Thus far, the ship-in-bottle strategy is the main developed encapsulation method for the construction of MOF composites in which metal-organic frameworks (“bottles”) are first created. Subsequently, precursors are incorporated inside the cavity of pre-formed MOFs through diffusion to then form nanocrystals under certain conditions. Therefore, nucleation and growth occur within MOF pores. Generally, the process involves two separate steps: loading of the perovskite precursor into the pores and nucleation followed by growth of perovskite nanocrystals inside the pores [134]. This strategy has been the preferred choice for confining ultrasmall (subnanometer) NCs within MOF pores over the rest of the strategies due to (i) the immobilizing principle, (ii) the simple experimental procedure, (iii) the possibility of yielding surface-clean composites, and (iv) avoiding overcoming the high interfacial energy barrier between MHPs and MOFs [135]. 

The ship-in-bottle strategy can further be classified into four categories, namely sequential deposition, in situ deposition, direct conversion, and physical mixing (Figure 2a–d). 

Sequential deposition is the most used approach since it allows for the incorporation of precursors sequentially, as illustrated in Figure 2a. Once precursors are located in the MOF cavities, MHPs will tend to form inside. By this approach, ultraconfined MHPs in MOF cages can be achieved and stabilized from aggregation or leaching [101]. Other works also reported the sequential deposition for synthesizing MHP@MOF composites [102,103,104,105,107,114,119,120,123,124,126,130,133]. Some of them have been carried out without solvent [109,125,131], making this a more sustainable methodology. Nevertheless, there are still some important aspects that need to be addressed: (i) the stoichiometric ratio of perovskite precursors inside pores, owing to the variability of MOF templates, that result in deep trap states for non-stoichiometric perovskites; (ii) MOF framework mechanical stressing due to the growth of perovskite nanocrystals inside the pores and their intrinsic stability under MHP synthesis conditions; and (iii) the spatial distribution of perovskite nanocrystals related to the large diffusion resistance of the perovskite precursors into MOF pores. Therefore, the loading yield of perovskite nanocrystals is formed.

In situ deposition, also known as the pore-encapsulated solvent-directed (PSD) approach, is the easiest way to form MHP@MOF composites, wherein all building block precursors of both MHPs and MOFs are mixed, as illustrated in Figure 2b. Here, the MOF host is first built, in which perovskite precursors are embedded inside pores resulting in a suspension. Thereafter, a considerable amount of a “bad solvent” as toluene is added to this resulting suspension, giving rise to nucleation and growth of emissive MHPs inside the matrix under ambient conditions [121,128]. This is an attractive approach, owing to its scalability and ability to save time, energy, and costs, and it shows that the composite has high robustness to different conditions such as moisture, air, solvents, temperature, and UV/visible light. In addition, this strategy was also applied to the formation of MHPs in xerogels (MOG), although more severe conditions were needed [108]. In contrast to the above-mentioned sequential deposition approach, this strategy may tend to degrade the crystalline structure as long as the formed perovskite size is larger than the MOF cavity, creating, in this way, such structural defects on the MOF host, damaging the optical and electronic properties of MHPs to some extent [128]. Additionally, organic linkers or solvents need functional groups to trap the precursor NCs inside and stabilize the NCs formed.

The direct conversion approach uses an MOF as a sacrificial template to grow MHPs inside the cavity as illustrated in Figure 2c. This approach was developed to palliate the large diffusion resistance that hinders the accessibility of perovskite precursors into the MOF host, and hence it provides a simple and fast strategy to integrate perovskite nanocrystals into the MOF matrix, as it reduces the number of steps. In this regard, MOFs with the cations MHP precursors in the framework first need to be synthesized. Subsequently, the metal halide source is integrated into the pores to lead to partial decomposition to produce MHP nanocrystals. Based on the conversion approach, this strategy can be employed for confidential information protection since the starting MOF is invisible and then is converted into a bright luminescent composite [122]. Similar to the above-mentioned method, the formation of a larger-sized perovskite nanocrystal inside pores during the nanocrystal growth could result in the partial degradation of the MOF matrix, giving rise to a slight aggregation of perovskite nanocrystals to some extent, and the final optical properties might be affected.

The physical mixture approach is based on the simple mixture of both the preformed MHP nanocrystals and MOFs under sonication or stirring, as illustrated in Figure 2d. One of the limitations of this technique is the need to use appropriate MOF pores able to integrate perovskite nanocrystals. However, thanks to the ability of post-treating MOFs, their pores can be enlarged by templating agents to expand the pore size and produce larger cavities [106] or even by using mesoporous MOFs, wherein presynthesized MHPs can be encapsulated through simple mixing [129]. Unfortunately, the size, shape, and structural composition of the final hybrids are not well controlled by this approach.

### 2.2. Bottle-around-Ship Method

The bottle-around-ship strategy, also known as the template synthesis approach, consists of the assembly of MOFs on the preformed MHP surface, as illustrated in Figure 2e. By using this strategy, MHPs are uniformly distributed throughout the MOF shell [127]. In this case, the issues associated with the aggregation of MHPs on the external surface are reduced, and the size, morphology, and structure of entrapped MHPs can be easily controlled because they are formed before the assembly with MOF precursors [136]. The main difficulty of this approach is preserving the crystalline structure of MHP nanocrystals under the synthesis conditions used to grow MOF shells, considering their low stability against harsh agents such as temperature, humidity, oxygen, and UV-light irradiation.

## 3. Properties and Applications of MHP@MOF Composites

Owing to the synergistic effect between MHPs and MOF matrixes, the resulting photoactive porous composites show better or even new properties and functionalities that can be effectively employed in several applications such as (i) lightning in light-emitting diodes, (ii) light-harvesting in solar cells, (iii) sensing in temperature sensor and analyte detection, (iv) encryption and decryption for confidential information, and (v) photocatalysis in degradation of organic pollutants and CO_2_ reduction. This section overviews and highlights the photoactive porous composites outlined in Table 1, according to their application. Most of the examples are photoactive composites based on MHP nanocrystals; however, bulk MHP materials have also been combined with MOF heterojunctions through a sequential solution-processed method (sequential spin coating) to produce perovskite solar cells (PSCs) with improved performance features, power conversion efficiencies, and stabilities (see Section 3.2, Perovskite Solar Cells) [110,111,112,113,114,115,116,117,118,119].

### 3.1. Light-Emitting Diodes

**Figure 3:**
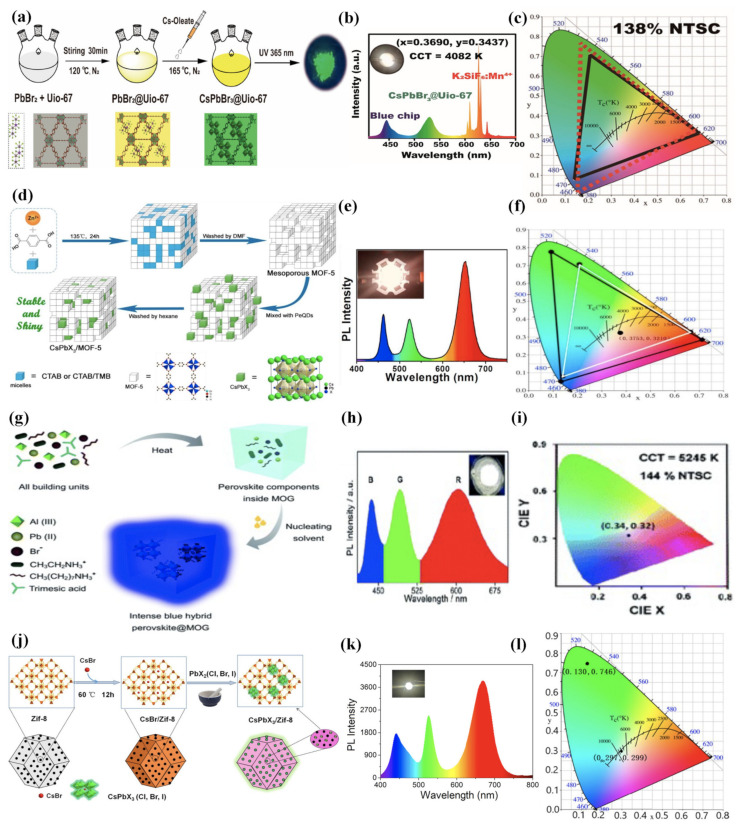
(**a**) Proposed synthesis procedure for the formation of typical CsPbBr_3_@UiO-67 composite and the corresponding structural models. (**b**) PL spectrum of the as-fabricated WLEDs with CsPbBr_3_@UiO-67 composite and commercial K2SiF6:Mn4+ red phosphor deposited on the blue-chip (InGaN, 455 nm). Inset: Photograph of the WLEDs (x: 0.3690, y: 0.3437). (**c**) CIE colour space coordinates of as-fabricated WLEDs showing a wide colour gamut (138% NTSC). Adapted with permission from ref. [105]. Copyright 2019, American Chemical Society. (**d**) Synthesis strategy of the mesoporous MOF-5 crystals and the CsPbX_3_/MOF-5 composites. (**e**) PL spectrum of the CsPbX_3_/MOF-5, red CsPbBr_0.6_I_2.4_/MOF-5, and the blue InGaN chip (black line) compared with the NTSC standard (white line). (**f**) CIE colour coordinates of the W-LED device. Adapted with permission from ref. [126]. Copyright 2018, Elsevier B.V. All rights reserved. (**g**) Schematic of synthesis procedure of intense blue hybrid perovskite@MOG nanocomposite. (**h**) The emission spectrum of white LED fabricated by a combination of blue-emitting EAPbBr_3_@MOF (B), green-emitting MAPbBr_3_@MOG (G), and red-emitting Mn(II)-doped EAPbBr_3_ NCs (R) deposited on a UV chip. Inset: shows a photograph of the white LED with an applied current of 20 mA. (**i**) CIE colour coordinates of the as-fabricated white LEDs (x: 0.34, y: 0.32). Adapted with permission from ref. [128] under Creative Commons Attribution-NonCommercial 3.0 Unported License. (**j**) Mechanochemical synthesis of CsPbX_3_/ZIF-8 composites using CsBr/ZIF-8. (**k**) PL spectrum and corresponding white LED picture of white CsPbX_3_/ZIF-8 composite prepared by mixing blue, green, and red-light emitting components. (**l**) CIE colour coordinates of LEDs. Adapted with permission from ref. [109]. Copyright 2021, American Chemical Society.

The remarkable photoluminescence properties, ease tunability of emission wavelength over the entire spectrum, high colour purity, and narrow full-width at half maximum of MHP nanocrystals make them amazing candidates for light-emitting diode (LED) applications [137]. The first example of MHP@MOF composites for LED applications was performed by Zhang et al. [105] in 2019 in which they successfully prepared CsPbX_3_@UiO-67 composites through a sequential deposition approach of perovskite precursors into a stable Zr-based MOF (UiO-67) matrix as shown in Figure 3a.

Inorganic MHP nanocrystals such as CsPbBr_3_ and CsPbBr_1.2_I_1.8_ were grown inside different types of UiO-67 channels, yielding a photoactive porous composite with excellent photoluminescence properties and enhanced stability. A white LED was fabricated by using green-emitting CsPbBr_3_@UiO-67, commercial red phosphors (K_2_SiF_6_:Mn^4+^), and an InGaN blue chip with a correlated colour temperature of 4082 K (Figure 3b) and 138% of the National Television Standards Committee (NTSC) standard, which indicated that composites have potential applications in display fields as shown in Figure 3c. Shortly after, Ren et al. [106] successfully embedded CsPbX_3_ NCs into mesoporous MOF-5 crystals by a simple physical mixture of both components as shown in Figure 3d. By using surfactant cetyltrimethylammonium bromide (CTAB) and 1,3,5-trimethylbenzene (TMB) as templating agents, they were able to expand the MOF-5 from microporous to mesoporous size to incorporate MHP nanocrystals with a size of about 10 nm. The MOF host renders perovskite invulnerable to environmental conditions and anion exchange. The CsPbX_3_@MOF-5 composite resulted in enhanced stability (thermal-, photo-, and long-term stability), and the anion exchange was greatly suppressed while remarkable optical properties were preserved. Finally, a warm white LED was successfully fabricated by using green-emitting CsPbBr_3_@MOF-5, red-emitting CsPbBr_0.6_I_2.4_@MOF-5, and an InGaN blue chip (Figure 3e) with the correlated colour temperature of 3607 K along with a wide colour gamut that encompasses 124% of NTSC standards, which reflected excellent-quality warm white light with a high luminous efficiency of 21.6 lm/W, as shown in Figure 3f. Zhang et al. [107] reported the preparation of CH_3_NH_3_PbBr_3_@Bio-MOF-1 composites through a sequential deposition and investigated their optical properties and air stability. The fact that they used Bio-MOF-1 as a host matrix was due to diverse factors: (i) it presents abundant pores, which enables Bio-MOF-1 to encapsulate nano-sized MHPs; (ii) Bio-MOF-1 can strongly absorb metal ions including Pb^2+^, which is suitable for growing the perovskite inside; (iii) the host matrix has blue fluorescence, originating from aggregated BPDC linkers and the metal-ligand charge transfer-based luminescence within the MOFs; and (iv) the emission of the Bio-MOF-1 can be modulated by doping with guest luminescence materials. Consequently, a white LED with Commission Internationale del’Eclairage (CIE) colour coordinates (x: 0.32, y: 0.31) very close to the ideal value was fabricated by using bare Bio-MOF-1 (blue emission), MHP@Bio-MOF-1 (green emission), and ruthenium (II) complex doped Bio-MOF-1 (red emission). In the same year, Mollick et al. [108] reported for the first time the formation of a luminescent and stable composite through embedding CH_3_CH_2_NH_3_PbBr_3_ MHPs in a porous metal-organic gel (MOG) matrix via the in situ approach, as shown in Figure 3g, where all building blocks were mixed to first form the MOG matrix in which the perovskite precursors were trapped inside and then treated with toluene to obtain the corresponding CH_3_CH_2_NH_3_PbBr_3_@MIL-100(Al) composite. An unprecedented enhancement in the Φ_PL_ (up to 10 times) and photo and water stability was achieved for this composite. A white LED was fabricated by using blue-emitting CH_3_CH_2_NH_3_PbBr_3_@MIL-100(Al) combined with the green-emitting CH_3_NH_3_PbBr_3_@MIL-100(Al) composite and red-emitting Mn (II)-doped CH_3_CH_2_NH_3_PbBr_3_@MIL-100(Al) composite (Figure 3h), exhibiting a wide colour gamut of 144% of NTSC standard and CIE colour coordinates (x: 0.34, y: 0.32), as shown in Figure 3i. Ren et al. [126] designed a novel strategy based on the sequential deposition approach where the mesoporous indium-based MOF (ZJU-28) was first ion-exchanged with Cs^+^ using a CsX solution. Cs-ZJU-28 then reacted with PbX_2_ solution at high temperatures to give the corresponding CsPbX_3_@ZJU-28 composite with improved stability, which shows great promise in different applications. The dual emission is attributed to the emission of organic linkers in the host matrix and the excitonic emission of the perovskite. Thanks to this dual-emission of the CsPbX_3_@ZJU-28 composite and by altering the halide composition of the MHP nanocrystals, a white LED was fabricated with a correlated colour temperature of 3748 K and CIE colour coordinates (x: 0.38, y: 0.35), indicating a pure white emission. Recently, a zeolitic imidazolate framework-8 (ZIF-8) was employed by Zhao et al. [109] to encapsulate CsPbX_3_ NCs through a mechanochemical strategy using a sequential deposition approach, as shown in Figure 3j, thus considerably improving the photo and storage stability and resistance to ion-exchange of CsPbX_3_ NCs. An energy transfer from ZIF-8 to MHPs improved the emission of the perovskite, as expected due to the overlapping of the perovskite absorption with the broad emission band of ZIF-8 from 360 to 600 nm, yielding a Φ_PL_ = 72%. Moreover, a white LED was fabricated (Figure 3k) with CIE colour coordinates (x: 0.30, y: 0.30) and lumen efficiency of 11.5 lm/W, as shown in Figure 3l. All of these examples demonstrated the potential of photoactive porous composites based on MHP for LED technology.

### 3.2. Perovskite Solar Cells

The challenge of perovskite solar cells (PSCs) is the long-term phase stability against working conditions that hamper their commercialization. For that reason, MOFs have been established to amend these issues. MOFs can also be solution-processed, and the optoelectronic properties (bandgap) can be tuned by controlling the MOF constituents, being used in a variety of ways in PSC devices, such as charge transport material, additives in charge transport materials, scaffolds in perovskite solution, and interlayers.

**Figure 4:**
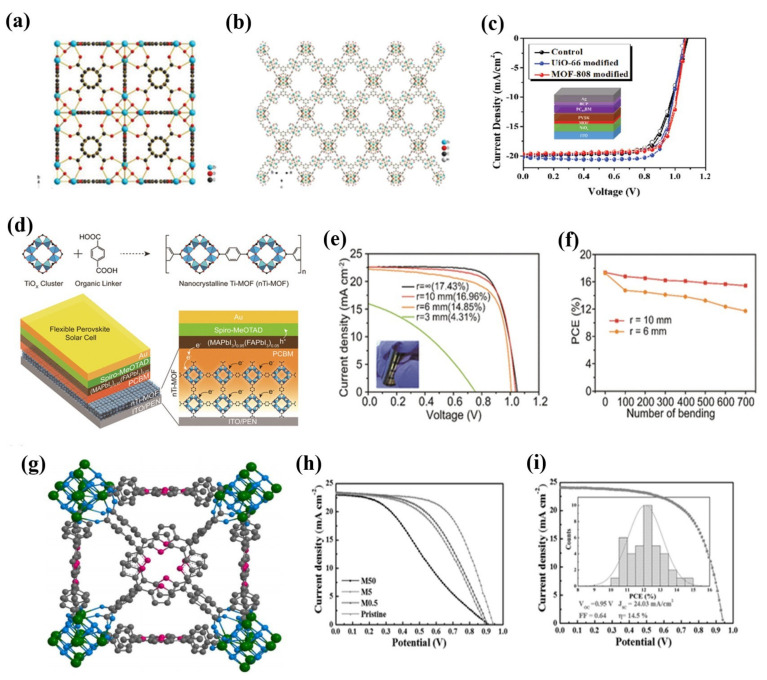
(**a**) The crystal structures of UiO-66 and (**b**) MOF-808 are illustrated. (**c**) The J-V curves of the MOF-modified devices measured under 1 sun. Adapted with permission from [114] under Creative Commons CC BY License. (**d**) Schematic of the synthesis protocol for nanocrystalline Ti-MOF (nTi-MOF). The successively linked TiO_2_ clusters with BDC molecules create small nanocrystals. The device structure of a flexible perovskite solar cell incorporating nTi-MOF/PCBM ETL. The magnified image indicates electron and hole transfer from the perovskite toward interlayers, nTi-MOF/PCBM and spiro-MeOTAD, respectively. (**e**) J-V curves of the flexible devices measured from r = ∞ to r = 3 mm and (**f**) bending test of the devices for r = 10 mm and r = 6 mm up to 700 bending cycles. Adapted with permission from [113]. Copyright 2018, American Chemical Society. (**g**) Crystal structure of MOF-525. Is illustrated (**h**) J-V curves of the perovskite and MOF/perovskite solar cells and (**i**) J-V curve of the best M5 cell. Inset: histogram of average PCE values of 40 devices. Adapted with permission from [111]. Copyright 2015, WILEY-VCH Verlag GmbH & Co. KGaA, Weinheim.

MOFs provide excellent electron and hole transport paths and are effective in suppressing charge recombination by improving the quality of perovskite films [32]. In particularly, the MOF/perovskite heterojunction has additional advantages against humidity and chemical stabilities [44]. It is worth noting that perovskites used in this field are based on bulk materials unless specified. The first work in which a MOF matrix was integrated into a PSC was done by Vinogradov et al. [110] in 2014 as a heterojunction system in which they synthesized, in a single step, the MIL-125@TiO_2_ composite to produce a depleted perovskite/MIL-125@TiO_2_ heterojunction solar cell, strengthening the charge recombination suppression at the interface. A PSC produced V_OC_ = 0.85 V and I_SC_ = 10.9 mA/cm^2^, corresponding a PCE of 6.4%. Moreover, the MIL-125@TiO_2_-based heterojunction PSC presented durable stability over time (up to 30 days) under ambient atmosphere. Years later, Shen et al. [112] improved the film quality through interfacial engineering between perovskite and electron-transporting layers (ETL). They were the first to use ZIF-8 as an interlayer between mesoporous (mp)-TiO_2_ and perovskite layers to control the growth of the hybrid perovskite crystal layer and produce an enhancement in the photovoltaic performance, resulting in a higher PCE of ca. 17% in comparison to that exhibited by mp-TiO_2_ PSC (14.75%). It was confirmed that the ZIF-8 interlayer inhibited the recombination of photogenerated carriers at the interface and improved charge extraction. They also investigated the ZIF-8 coating time effect on the morphologic properties of the perovskite layer. Simultaneously, the increased grain sizes and the reduced grain boundaries based on the ZIF-8 coating layer can improve the quality of the perovskite film. Similarly, Lee et al. [114] reported the use of two types of Zr-MOFs, UiO-66 and MOF-808, as interlayers to prepare efficient and stable inverted p-i-n PSCs, as shown in Figure 4a,b. They performed as a UV filter and enhanced perovskite crystallinity, yielding PCEs of 17.01 and 16.55% for UiO-66 and MOF-808, respectively, as shown in Figure 4c. Other works based on the use of MOFs as interfacial layers in PSCs are reported elsewhere [117,118]. Ryu et al. [113] designed Ti-based MOF NPs as electron-transporting materials in flexible mixed cation-PSCs at an ambient temperature, as shown in Figure 4d. It was observed that when [6,6]-phenyl-C61-butyric acid (PCBM) was added, the conductivity of the MIL-125(Ti) improved considerably, and the microcracks on the MIL-125(Ti) film were filled. Thereby, the PCE of 16.41 improved up to 18.94% using PCBM. Remarkably, flexible PSCs with higher PCEs of up to 17.43% (Figure 4e) were obtained, and the durability was maintained over 700 bending cycles (15.43%), as shown in Figure 4f. Similarly, Co-doped TiO_2_ was synthesized through the solvothermal method and used as effective electron-transporting material in PSCs since co-doping promoted electron transport and lowered charge recombination, giving rise to a PCE of 15.75% with an open-circuit voltage of 1.027 V, a current density of 24.078 mA/cm^2^, and a fill factor (FF) of 64.95% [116]. MOFs have been used as doping in the hole-transporting layers (HTL). For instance, Dong et al. [115] reported well-dispersed polyoxometalate@MOF composites (POM@Cu-BTC) in the extensively used spiro-OMeTAD HTL, controlling the oxidation, improving the stability of the HTL (maintaining ca. 90% of the initial PCE value after long-term storage in ambient conditions), and achieving higher PCEs than undoped material (21.44% vs. 20.21%), due to the increased electron affinity and oxidation potential of the composite. Another way to use MOFs is as a porous scaffold. In 2015, Chang et al. [111] incorporated microporous Zr-based porphyrin MOF (MOF-525) NCs, as shown in Figure 4g, with a size of about 140 nm as additives into the perovskite precursor solution to be deposited, thus improving the morphology and crystallinity of the resultant perovskite thin film. The microporous scaffold provided an ordered arrangement of perovskite crystallites during the initial stage of crystallization, and finally a PCE of up to 12% was obtained, as shown in Figure 4h. The histogram of the average PCEs measured from 40 devices is shown in Figure 4i. Lee’s work [114] also reported MOFs as a scaffold for the nucleation of perovskite films. The perovskite/Zr-MOF heterojunction improved the crystallinity of the perovskite film. Moreover, the passivation of defects enhanced the properties of perovskite as well as the resistance of the film to moisture penetration, improving the PCE up to 18.01 and 17.81% for hybrid perovskite/UiO-66 and hybrid perovskite/MOF-808 PSCs, respectively. Recently, the effect of different additives of Zr (IV)-, In (III)-, and Zn (II)-MOFs in the perovskite layer to control the crystallization process and film formation was investigated [119]. The additives yielded a better perovskite morphology with fewer voids between perovskite grains. Although the PCE was quite low (2.95%), it could be improved to 5.64% (ca. 91% enhancement) after adding 2 wt% of Zn (II)-MOF to the perovskite solution.

### 3.3. Sensing Applications

Due to the appealing characteristics of MHPs, their use in sensing applications has attracted tremendous research interest in recent years, in particular due to their ability to monitor both emission intensity and emission lifetime for ion-detection [120] and non-contact temperature sensing behaviours [120,121,126]. Nevertheless, even though the remarkable performance of MHPs has been demonstrated, their practical applications are still greatly hindered by the inherent instability discussed in previous sections [138]. 

**Figure 5:**
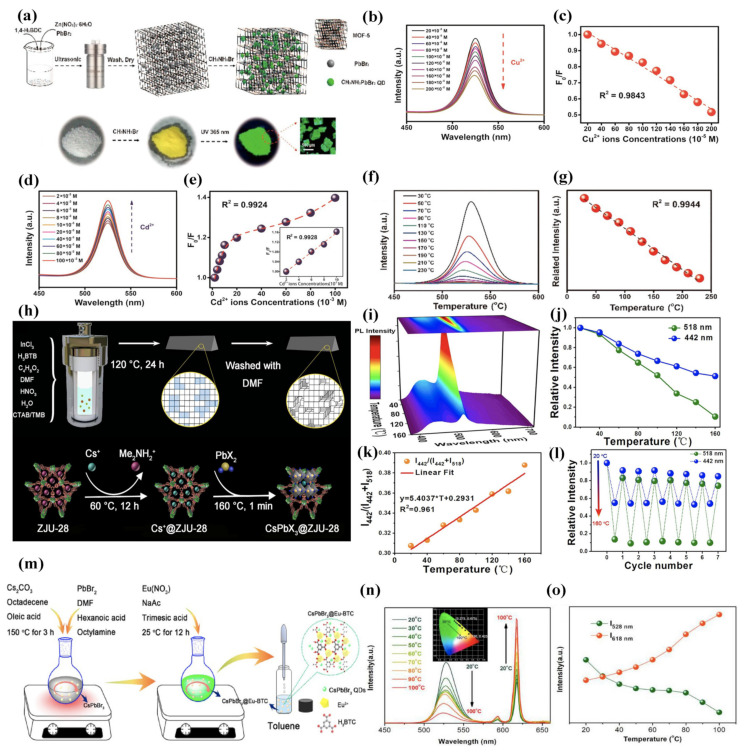
(**a**) Schematic diagram of the two-step approach to CH_3_NH_3_PbBr_3_@MOF-5 composites. The first step included the synthesis of PbBr_2_@MOF-5 precursor by a solvothermal method, and the second step involved the addition of CH_3_NH_3_Br solvent for the final CH_3_NH_3_PbBr_3_@MOF-5. Illustrative images of the PbBr_2_@MOF-5 precursor, the CH_3_NH_3_PbBr_3_@MOF-5 composite (under daylight and 365 nm UV-light excitation) and its fluorescence microscope morphology. (**b**) PL spectra of CH_3_NH_3_PbBr_3_@MOF-5 composites depending on various Cu^2+^ ion concentrations. (**c**) Stern–Volmer plot for the F_0_/F values and different Cu^2+^ ion contents. (**d**) PL spectra of CH_3_NH_3_PbBr_3_@MOF-5 composites depending on various Cd^2+^ ion concentrations and (**e**) Stern–Volmer plot for the F_0_/F values and different Cd^2+^ ion contents. (**f**) PL spectra of CH_3_NH_3_PbBr_3_@MOF-5 composites depending on increasing temperature from 30 to 230 °C and (**g**) variation of the emission intensity depending on temperatures and a linear fitting. Adapted with permission from [120]. Copyright 2018, American Chemical Society. (**h**) Schematic illustration of the synthesis strategy of the mesoporous ZJU-28 and representative CsPbX_3_@ZJU-28. (**i**) The temperature-dependent PL spectra of CsPbBr_3_@ZJU-28 in 3D colour mapping surface with projection and (**j**) the relative emission intensity with temperature increases for the excitonic emission (518 nm) and the π-π* transitions (442 nm) of the CsPbBr_3_@ZJU-28 composite. (**k**) The plots of the luminescence intensity of I_442_/(I_442_ + I_518_) versus temperature and (**l**) temperature cycle test of the CsPbBr_3_@ZJU-28 composite between 20 and 160 °C. Adapted with permission under [126]. Copyright 2019, Elsevier B.V. All rights reserved. (**m**) Schematic illustration for the preparation process of CsPbBr_3_@Eu-BTC. (**n**) Temperature-dependent PL spectra of CsPbBr_3_@Eu-BTC in the temperature range of 20–100 °C excited at 339 nm (inset: the CIE (x,y) coordinate diagram of emission colours at various temperatures) and (**o**) integrated emission intensities of CsPbBr_3_@Eu-BTC QDs and Eu^3+^: ^5^D_0–_^7^F_2_ versus various temperatures. Adapted with permission from [121]. Copyright 2020, American Chemical Society.

In 2018, Zhang et al. [120] reported the encapsulation of CH_3_NH_3_PbBr_3_ NCs into the pores of MOF-5 microcrystals by a sequential deposition approach, as shown in Figure 5a, to prepare CH_3_NH_3_PbBr_3_@MOF-5 composites which exhibited excellent water resistance and thermal stability for selective and sensitive metal ion detection in an aqueous solution over a wide pH range (3–11). Plenty of metal ions including Al^3+^, Bi^3+^, Co^2+^, Cu^2+,^ and Fe^3+^ significantly quenched the perovskite emission (Figure 5b), whereas, for the Cd^2+^ ion, the emission increased. In particular, the Stern–Volmer (SV) analysis with a Cu^2+^ addition (Figure 5c) indicated the presence of static quenching of the emission, as opposed to what happened in the case of the Cd^2+^ addition (Figure 5d,e), wherein the enhancing phenomenon of the emission could be attributed to a combination of dynamic and static enhancement, related to the stabilization effect on the composite by Cd^2+^ ions. This demonstrated that the composite can be employed as stable and versatile fluorescent sensors for detecting several metal ions in an aqueous solution over a wide range of pH values, excluding strong acid (pH 1) and strong base (pH 13) conditions. This system can be used for a selective and sensitive detection of Cu^2+^ with a linear Stern–Volmer response (R^2^ = 0.9843) in the concentration range from 20 to 200 × 10^−5^ M. Additionally, this composite showed temperature-dependent emission properties, in which perovskite emission was substantially quenched with increasing temperature from 30 to 230 °C, as shown in Figure 5f, thus allowing a linear relationship between emission intensity and temperature to be built that can be used as a ratiometric thermometer for temperature monitoring, as shown in Figure 5g.

On the other hand, Ren et al. [126] reported the preparation of CsPbX_3_@ZJU-28 composites by a sequential deposition approach in which Cs^+^ cations were first incorporated in the mesoporous blue-emitting In-based MOF (ZJU-28) and then reacted with PbX_2_ to yield the corresponding CsPbX_3_@ZJU-28 composite at high temperatures, as shown in Figure 5h. This composite presented dual-emission, the blue broadband (442 nm) from π-π* transitions of organic ligands in ZJU-28, and the narrow band from excitonic emission of CsPbX_3_ (518 nm). This composite was used in diverse applications such as anti-counterfeiting, temperature sensing, and LEDs due to both excitation-wavelength and temperature-dependent emission properties, improved stability of MHPs, and higher resistance to ion-exchange facilitated by the ZJU-28 matrix. The total emission intensity of the CsPbBr_3_@ZJU-28 composite decreased following the increasing temperature from 20 to 160 °C, as shown in Figure 5i, decreasing the green emission faster than the blue one as a consequence of the different temperature tolerance of CsPbBr_3_ QDs and the ZJU-28 matrix (Figure 5j). The variation of luminescence intensity [I_442 nm_/(I_442 nm_ + I_518 nm_)] as a function of temperature (range 20–160 °C) showed a good linear relationship with a correlation coefficient of R^2^ = 0.961 as shown in Figure 5k, which further verifies its use as a thermosensor. Furthermore, the variation of the excitonic emission and the π-π* emission intensities of the CsPbBr_3_@ZJU-28 at 20–160 °C for several cycles is shown in Figure 5l, showing that both the excitonic emission and the π-π* transition of the CsPbBr_3_@ZJU-28 have the stable reversible property, which results in the CsPbBr_3_@ZJU-28 having a great opportunity in temperature sensing applications. Another work by Liu et al. [121], in which the authors designed a novel self-calibrated optical luminescent thermometer of a CsPbBr_3_@Eu-BTC composite, was reported the following year. The composite was prepared by an in situ approach in which MHPs were encapsulated inside Eu-BTC pores, as shown in Figure 5m, exhibiting two main bands associated with green emission of CsPbBr_3_ NCs (528 nm) and red emission attributed to Eu^3+^ (618 nm). The effect on the emission intensity of those bands in the temperature range of 20–100 °C was investigated, showing a different behaviour, i.e., the green-emitting band quenched, and, conversely, the red-emitting band increased the emission intensity, as shown in Figure 5n. The fluorescence intensity ratio of both bands in this composite (I_618 nm_/I_528 nm_) allows its use as a ratiometric thermometer for accurate temperature monitoring applications with a maximum relative sensitivity of 3.9% °C^−1^ at 20 °C and an excellent temperature resolution of 0.004 °C, indicating its superiority to serve as a nanothermometer for optical temperature sensing, as shown in Figure 5o.

### 3.4. Information Security

At present, new techniques are required to show a high-security level in our daily lives [139]. Particularly, highly photoluminescence porous composites are suitable candidates for being used as encryption/decryption and anti-counterfeiting tools in the field of information security, thanks to the readily on/off switching of their emission by reversible destruction-generation or phase change in MHPs and fixation of precursors conferred by the MOF porous matrix.

**Figure 6:**
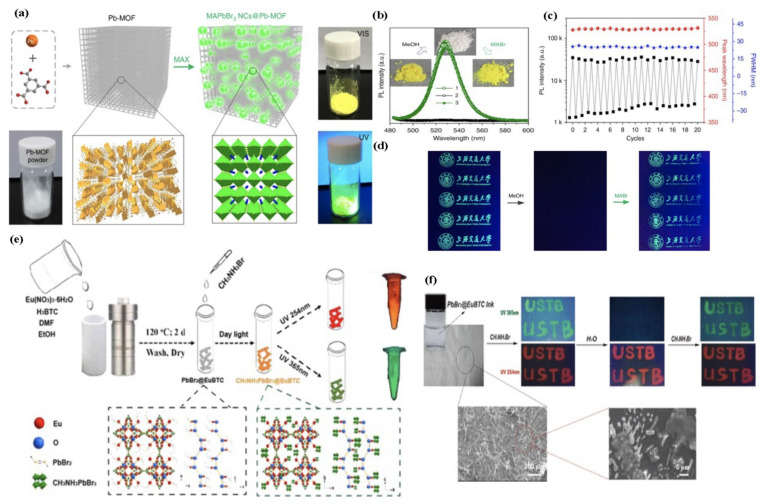
(**a**) Schematic of the conversion process. MAX represents the halide salt (CH_3_NH_3_X, X = Cl, Br, or I). The green spheres in the matrix represent the MAPbBr_3_ NCs. The two black boxes show 3D crystal structure of the Pb-MOF (left) and MAPbBr3 (right). The Pb coordination polyhedra (the Pb atoms are coordinated by nine O atoms, in which two O atoms of one carboxylate coordinate to a Pb and also bridge two adjacent Pb atoms) and MAPbBr_3_ is represented in orange and green, respectively. Other atom colour schemes: C = grey, O = red, N = blue, Br = yellow. H-atoms have been omitted for clarity. Optical images of MAPbBr_3_ NCs@Pb-MOF powder under ambient light and 365 nm UV lamp. (**b**) Sequential optical images and PL emission spectra of MAPbBr_3_ NCs@Pb-MOF after one cycle of the impregnation-recovery process; 1, 2, and 3 represent the original, impregnated, and recovered powder sample of MAPbBr_3_ NCs@Pb-MOF, respectively. (**c**) PL intensity, peak wavelength, and FWHM of MAPbBr_3_ NCs@Pb-MOF in the impregnation-recovery cycles as a function of cycle number. (**d**) Reversible fluorescence switching of the MAPbBr_3_ NCs@Pb-MOF pattern in one encryption–decryption cycle (methanol impregnation for encryption and MABr spraying for decryption). Adapted with permission from [122] under a Creative Commons Attribution 4.0 International License. (**e**) Schematic illustration targeted the two-step fabrication of CH_3_NH_3_PbBr_3_@EuBTC composites, including (1) the PbBr_2_@EuBTC precursor prepared by a solvothermal method and (2) CH_3_NH_3_PbBr_3_@EuBTC composites precipitated with the addition of CH_3_NH_3_Br solution. The two dotted frames give the crystal structures of the PbBr_2_@EuBTC and CH_3_NH_3_PbBr_3_@EuBTC along the b and c axes. (**f**) Reversible fluorescence switching of the USTB pattern written on a paper at different stages and different excitation wavelengths of 254 nm and 365 nm. SEM images of USTB pattern on a paper at 200 μm and 5 μm. Adapted with permission from [123]. Copyright 2018, American Chemical Society.

For instance, the first work involving the direct conversion of an invisible Pb-based MOF into luminescent CH_3_NH_3_PbX_3_@Pb-MOF composites for confidential information encryption and decryption and storage application was reported by Zhang et al. [122] in 2017. The initially invisible Pb-MOF was employed as the sacrificial porous template through direct conversion of the Pb-MOF into MHPs inserted in MOFs when treated with a CH_3_NH_3_Br solution, as shown in Figure 6a, producing bright luminescent materials when printing in diverse patterns. Confidential information can be encrypted in the Pb-MOF pattern, and after its conversion into an emissive composite, the effective information decryption can be achieved. MHPs in the MOF matrix were destroyed by polar solvent impregnation (Figure 6b), thus quenching the luminescence of the material and giving up to 20 information encryption and decryption cycles with a negligible decrease in emission intensity, as shown in Figure 6c. Reversible fluorescence switching of the MAPbBr_3_ NCs@Pb-MOF pattern in one encryption–decryption cycle was observed (Figure 6d). Zhang et al. [123] reported on the preparation of CH_3_NH_3_PbBr_3_@Eu-BTC composites as an anti-counterfeiting tool through a sequential deposition approach, as shown in Figure 6e. It was observed that the composite presented three outstanding emission bands, i.e., at 513, 593, and 617 nm, when excited at 365 and 254 nm, corresponding to the excitonic emission of MHP and those attributed to the Eu-BTC emission. The PbBr_2_ salt was firstly incorporated in the MOF. Then, when treated with the CH_3_NH_3_Br solution and water, the MHP with green emission formed and degraded as a consequence of the crystalline structure destruction in presence of polar solvents. However, the red emission attributed to the Eu-BTC persisted, as shown in Figure 6f, suggesting a great potential in multimodal optical anti-counterfeiting applications. Other works with a similar application are reported elsewhere [124,125,126].

### 3.5. Photocatalysis

The development of green, sustainable, and cost-effective chemical processes spanning from hydrogen production [140] and CO_2_ conversion [141] to organic transformations for fuels or value-added chemicals [142], together with the degradation of dyes or pollutants [143], includes ongoing investigations using semiconductors. MHP nanocrystals are being exploited as photocatalysts owing to their strong light absorption capacity, efficient charge transport properties, and appropriate redox ability for target reactions [144]. For instance, CsPbBr_3_ nanocrystals were successfully used for the C-C coupling reaction of alkyl bromides, at room temperature under visible light irradiation, through the efficient pre-concentration of the substrate on the surface of the nanocrystals, assisted by the capping ligands [145]. Nonetheless, stability issues associated with the intrinsic nature of MHPs in polar solvents hamper the progress in this field in an aqueous medium [146]. 

**Figure 7:**
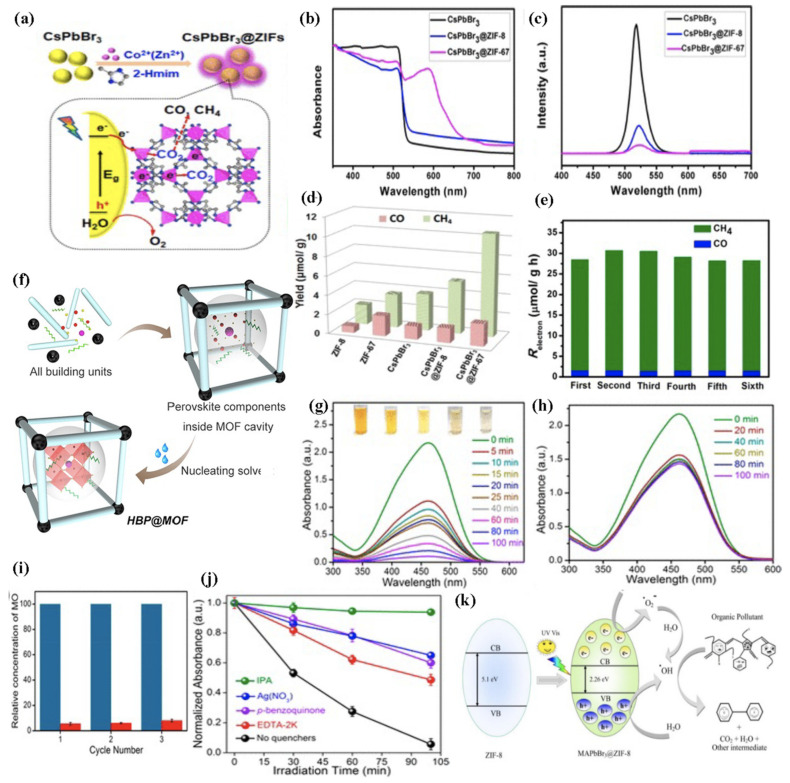
(**a**) Schematic illustration of the fabrication process and CO_2_ photoreduction process of CsPbBr_3_/ZIFs. (**b**) UV-vis absorption spectra and (**c**) steady-state PL spectra. (**d**) Product yield after 3 h of photocatalytic reaction and (**e**) recycling test of CsPbBr_3_@ZIF-67 6 times with CO_2_ refilled every 3 h. Adapted with permission from ref. [127]. Copyright 2018, American Chemical Society. (**f**) Pore-Encapsulated Solvent-Directed (PSD) procedure for preparing HBP@MOF composites. (**g**) Absorption spectra changes of MO solution degraded by MAPbBr_3_ composite under visible light irradiation for different time intervals. The inset photograph shows degradation MO by composite for 0, 10, 25, 60, and 100 min (from left to right) and (**h**) degradation of MO by ZIF-8 under visible light. (**i**) Recyclability test for MAPbBr_3_ composite after photocatalytic degradation. The blue colour denotes the concentration of MO molecules before photocatalysis reaction, and the red colour denotes the concentration of MO molecules after photocatalysis reaction and effects of different quenchers for degradation of MO by the composite under visible light. (**j**) The degradation of MO is much prevented for IPA solution (green line), a widely used hydroxyl radical scavenger, confirming that hydroxyl radicals produced during the process are mainly responsible for photocatalytic dissociation of MO. (**k**) Schematic of the photocatalysis reaction mechanism. Adapted with permission from [128]. Copyright 2019, American Chemical Society.

Kong et al. synthesized core@shell CsPbBr_3_@ZIF-(8,67) through the bottle-around-ship approach by directly growing MOF precursors on the MHP surface for photocatalytic CO_2_ reduction to CO and CH_4_, as shown in Figure 7a [127]. The resulting composite showed a largely improved moisture stability, CO_2_ capturing ability, and charge separation efficiency. For the synthesis of the core@shell composite, mild conditions were required to grow the MOF shell around the MHP surface, preserving the perovskite performance.

An obvious broadened visible-light absorption was observed for the CsPbBr_3_@ZIF-67 sample, as shown in Figure 7b, and ZIF-67 also quenched the perovskite emission to a greater extent compared to ZIF-8, as shown in Figure 7c, meaning that an efficient electron transfer between both systems was attributed to the active Co centres, which can serve as an electron reservoir to accept electrons to be activated. 

The composite catalysts exhibited enhanced CO_2_ reduction activity with electron consumption rates of 15.498 and 29.630 μmol/g·h for CsPbBr_3_@ZIF-8 and CsPbBr_3_@ZIF-67, respectively, which are 1.39- and 2.66-fold higher than pristine CsPbBr_3_, as shown in Figure 7d. Furthermore, after six cycles of catalytic activity, no obvious change in the electron consumption rate was obtained, indicating the important role of the ZIF shell, as shown in Figure 7e. Mollick et al. [128] prepared ultrastable composite materials based on a luminescent MHP@ZIF-8 composite, using two different organic cations in the perovskite, i.e., methylammonium and ethyl ammonium, through an in situ deposition approach (Pore-Encapsulated Solvent-Directed, PSD procedure), as shown in Figure 7f. All building blocks were mixed in situ, and after stirring, a white precipitate was observed as ZIF-8 particles were formed. Perovskite precursors were trapped inside and MHP nanocrystals were obtained after the addition of an excess of toluene. The photoactive porous composites showed outstanding chemical stability (90 days over a wide range of solvents), thermal stability (heated at 140 °C for 20 days), and photostability (continuous UV-light irradiation for 20 days). In addition, this composite was used as a heterogeneous photocatalyst to degrade toxic organic pollutants of dyes (methyl orange (MO) and methyl red (MR)) and antibiotics such as nitrofurazone (NFZ) directly in aqueous media. The CH_3_NH_3_PbBr_3_@ZIF-8 composite efficiently degraded MO within 80 min, as shown in Figure 7g, and no degradation was observed with MOF alone (Figure 7h). They also tested the recyclability of the composite and observed that more than 90% degradation was maintained even after three consecutive cycles, as shown in Figure 7i. To demonstrate the degradation mechanism, they performed the photocatalytic reaction individually using different scavengers, i.e., AgNO_3_ (radical scavenger), EDTA-2K (h^+^ scavenger), isopropyl alcohol (IPA, ·OH scavenger), and *p*-benzoquinone (O_2_^−^ scavenger), as shown in Figure 7j. It was demonstrated that the degradation mechanism followed in situ hydroxyl radical generation, as shown in Figure 7k. The reduction of CO_2_ was also studied with the MHP@MOFs composite using iron-based PCN-221(Fe_x_) MOFs and UiO-66(NH_2_) matrix [129,130].

## 4. Conclusions, Challenges and Perspectives

The design and preparation of novel photoactive porous composites have been the focus of research due to new and enhanced properties established in the composite that cannot be achieved by the individual components. 

The outstanding optical properties of MHPs, such as their large absorption coefficient, high carrier mobilities, high photoluminescence, and high defect tolerance, together with their low-cost precursors and easy fabrication, make them very promising materials in semiconductor-based technologies. Although there has been a significant amount of research progress on perovskite nanocrystals from the synthetic point of view and their optical properties, there are some challenges that need to be overcome to bring them into real applications. Increasing the MHP stability will boost their optoelectronic performance, broadening their great potential in photocatalysis and photoluminescence-related applications beyond the photovoltaic potential. The integration of MHP into porous MOFs can be considered a robust surface protection strategy, which offers remarkable stabilization towards external factors such as humidity. Compared to conventional encapsulation matrixes, multifunctional MOFs can be well designed as host matrices by varying organic linkers and metal nodes, which endow the MOF with structural diversity, high porosity, and high specific surface area.

This review highlighted the recent progress in the preparation of photoactive porous composites that afford significant insights for the future fabrication of advanced multifunctional materials. The most used methodology to prepare MHP@MOF composites was the ship-in-bottle by sequential deposition approach, in which MHP nanocrystals and ultra-confined MHP can be formed inside the pores. Moreover, the reaction can be done in the absence of solvents, making it a more sustainable method.

Regarding the thin-film technology, the main role of supramolecular MOFs concerns the perovskite grain crystallinity, film formation, and photovoltaic device performance. They have been used as an additive interlayer material between the absorber and transporting layers or as terminated agents, improving stability towards moisture, decreasing surface defects, and giving rise to superior optoelectronic properties (PCE up to ca. 21% in the case of 2D MOF-modified perovskite). 

The photocatalytic activity of the photoactive porous composite can be improved by the reduction of the electron-hole recombination of the MHP nanocrystals inside the MOF matrix, in which composition engineering can be used to tune the bandgap and therefore the photoluminescence properties of MHP. Double perovskites present a high bandgap, absorbing high-energy photons and having good photocatalytic responses. However, further stability improvement is required to increase their photocatalytic potential, and their integration into an MOF matrix could be a promising alternative. Effective harvesting of photons in a wide range of energies is key to improving the photocatalytic activity of MHP under sunlight. In order to provide visible and near-infrared emission, they can be doped with lanthanides ions that can be incorporated into the crystalline structure, broadening their applications.

Exciting emissive properties acquired in the composite due to the synergistic effect between MHP and MOFs extend their applications towards sensing, LED technology, and encryption/decryption. White LEDs were successfully achieved from MHP@MOFs composites based on inorganic and hybrid MHPs, exhibiting a wide colour gamut of 124 and 144% of the NTSC standard. 

The main challenge in the preparation of MHP@MOF composites lies in the synthesis approach used. In the case of the ship in a bottle, it is highly desired to improve the diffusion of the MHP precursors along the pore and the homogeneity in the precursor’s concentration to avoid nonstoichiometric nanocrystals and to preserve the MOF integrity during the MHP crystallization. When MHPs act as a template, interfacial nucleation should be controlled to avoid the mismatch between the crystal lattice of both components, reducing surface defects such as halide vacancies that directly affect the MHP emissive properties. Surface MHP engineering could facilitate the contact between MHP and MOFs, and therefore the correct selection of the organic ligands in the structure of MOFs plays a key role. In the one-step synthesis, the most promising approach is the mechanochemical preparation of the composite by mixing all precursors under solvent-free conditions. However, better control of the structural deformation of MOFs and MHP nanocrystal size distribution is required. The combination of MOF layers with semiconductor materials favours electron extraction and reduces electron-holes recombination, boosting the electron transport properties and photovoltaic performances of solar cells. MOFs can be incorporated into the light-absorber MHP layer, increasing the light-harvesting, but further studies are necessary to understand their role in the crystal growth and the reduction of defects in MHP, improving the PCE and stability of the composite. 

The development of deep-blue emissive and stable MHP@MOF composites is still a challenge. The exploration of other kinds of perovskite compositions and dimensionalities of the octahedral network seems to be a good alternative. The versatility of the MOF structure and easy chemical pore functionalization should be further explored to benefit the environment for MHP crystallization. Furthermore, theoretical studies could help to predict stable combinations of both materials with superior integrity and exciting properties.

The most promising research direction to boost the commercialization of LHP nanocrystal electroluminescent devices is to exploit their optical properties inside the composite and their chemical stability towards other external factors among humidity, such as light, heat, and oxygen. Further efforts are required for the development of brighter emissive composites in the whole range of the spectrum. Blue-emitting MHP nanocrystals usually present poor photoluminescence quantum yield and low chemical stability. In this sense, extremely narrow blue emission coming from controlled 2D perovskite material could be an excellent alternative for developing blue-emissive LEDs.

Even though the reported approaches are a good option to efficiently encapsulate MHPs inside the MOF matrix, the precise control of the size, morphology, and composition of incorporated NCs could be improved. Another concern of MHP nanocrystals is the toxicity of lead. Tin-based MHP nanocrystals present good emissive properties but suffer from stability issues due to the oxidation of Sn^2+^, so their incorporation into the matrix of MOFs could be an alternative to be explored. The preparation of emissive lead-free MHP nanocrystals, replacing lead with tin or germanium, will promote the design of environmentally friendly photoactive porous composites. 

Ongoing research on this new family of photoactive porous composites is still in its infancy and needs a joint effort from different disciplines such as material sciences, chemistry, photophysics, and others. The rational design of unprecedented multifunctional photoactive porous composites by the integration of MHP with different compositions, morphologies, and framework dimensionalities will promote the enhancement of photoluminescence efficiency and the exploration of new lines of research.

## Figures and Tables

**Table 1:** Summary table of the composition, synthesis methodology, structural and optical properties, and applications of the MHP@MOF composites.

MHP@MOF Composite	Synthesis Strategy	MOFs	Pore Size (nm) ^a^	MHP	MHP Size (nm)	λ_em_ (nm) ^b^	Φ_PL_ (%) ^c^	Application [Ref]
MAPbX_3_@HKUST-1	Ship-in-Bottle (Sequential Deposition)	HKUST-1 [Cu_3_(BTC)_2_, BTC = 1,3,5-benzene tricarboxylate]	1.66	MAPbI_2_Cl MAPbI_2_Br MAPbI_3_	1.5–2	536 655 715	- - -	Luminescence [101]
MAPbBr_3_@ZJU-28	Ship-in-Bottle (Sequential Deposition)	ZJU-28 (ZJU-28 = [In_3_(BTB)_4_](Me_2_NH_2_)_3_)	-	MAPbBr_3_	7.7 11	509 530	51	Multiphoton Exciton Luminescence [102]
CsPbX_3_@MIL-101	Ship-in-Bottle (Sequential Deposition)	MIL-101(Cr)	3	CsPbCl_3_ CsPbCl_2_Br CsPbCl_1_Br_2_ CsPbBr_3_ CsPbBr_2_I CsPbBr_1_I_2_ CsPbI_3_	1.14	417 426 454 520 569 633 698	- - - - - - -	Tunable Emission [103]
MAPbBr_3_@LnMOF	Ship-in-Bottle (Sequential Deposition)	LnMOF [Ln(tpob)(MDF)(H_2_O)]_n_, (Lntpob, Ln = Nd, Sm, Eu, Gd, Tb, Dy, H_3_tpob = 1,3,5-tris(4-carbonylphenyloxy)benzene)	-	MAPbBr_3_	-	515	-	Tunable Emission [104]
CsPbX_3_@UiO-67	Ship-in-Bottle (Sequential Deposition)	UiO-67	12 16	CsPbBr_3_ CsPbBr_1.2_I_1.8_	- -	521 634	39 30	Light-Emitting Diodes [105]
CsPbX_3_@MOF-5	Ship-in-Bottle (Physical Mixing)	MOF-5	25	CsPbBr_3_ CsPbBr_0.6_I_2.4_	9.49	519 655	52 56	White Light-Emitting Diodes [106]
MAPbBr_3_@Bio-MOF-1	Ship-in-Bottle (Sequential Deposition)	Bio-MOF-1 (Zn_8_(Ad)_4_(BPDC)_6_O·2Me_2_NH_2_, Ad = adeninate; BPDC = biphenyl-dicarboxylate)	-	MAPbBr_3_	-	519 (417,465)	16	Light-Emitting Diodes [107]
EAPbBr_3_@MIL-100(Al)	Ship-in-Bottle (in situ Deposition)	MIL-100(Al) (MOG)	2.5–10	EAPbBr_3_	3–11	436	53	Light-Emitting Diodes [108]
CsPbX_3_@ZIF-8	Ship-in-Bottle (Sequential Deposition) ^d^	ZIF-8	5.7	CsPbCl_3_ CsPbBr_3_ CsPbI_3_	5–10	- 520 -	51 72 57	Light-Emitting Diodes [109]
MAPbI_3_/MIL-125	-	MIL-125	-	MAPbI_3_	Bulk	-	-	Photovoltaic [110]
MAPbCl_x_I_3-x_/MOF-525	-	MOF-525	1.8	MAPbCl_x_I_3-x_	Bulk	-	-	Photovoltaic [111]
MAPbI_3_/ZIF-8	-	ZIF-8	-	MAPbI_3_	Bulk	-	-	Photovoltaic [112]
MA_0.95_FA_0.05_PbI_3_/MIL-125(Ti)	-	MIL-125(Ti)	-	MA_0.95_FA_0.05_PbI_3_	Bulk	-	-	Photovoltaic [113]
MAPbI_3_/UiO-66 MAPbI_3_/MOF-808	Ship-in-Bottle (Sequential Deposition)	UiO-66 (Zr_6_O_4_(OH)_4_(BDC)_6_) MOF-808 (Zr_6_O_4_(OH)_4_(BTC)_2_(HCOO)_6_)	1.3 1.9	MAPbI_3_	720 ^e^ 640 ^e^	-	-	Photovoltaic [114]
Cs_0.1_FA_0.747_MA_0.153_PbI_2.49_Br_0.51_/[Cu_2_(BTC)_4/3_(H_2_O)_2_]_6_	-	[Cu_2_(BTC)_4/3_(H_2_O)_2_]_6_	-	Cs_0.1_FA_0.747_MA_0.153_ PbI_2.49_Br_0.51_	Bulk	-	-	Photovoltaic [115]
MAPbI_3_/Co-doped-Ti-MOF	-	Co-Doped-Ti-MOF	6.79	MAPbI_3_	Bulk	-	-	Photovoltaic [116]
APbI_3_/ZIF-8	-	ZIF-8	-	(Cs/MA/FA)PbI_3_	Bulk	-	-	Photovoltaic [117]
APbI_3_/Zn-cbpp	-	2D Zn-cbpp [Zn(cbpp)(HCOO)]_n_, [hcbpp = 1-[4-carboxylbenzyl]-3-[pyrzin-2-yl]pyrazole]	-	(Cs/MA/FA)PbI_3_	Bulk	744	-	Photovoltaic [118]
MAPbBr_3_/X-LH_2_	Ship-in-Bottle (Sequential Deposition)	Zr-LH_2_ In-LH_2_ Zn-LH_2_ [(pydaH_2_)^2+^(pydc)^2−^, pyda = 2,6-pyridinediamine; pydcH_2_ = 2,6-pyridinedicarboxylic acid]	-	MAPbBr_3_	49 34 46	-	-	Photovoltaic [119]
MAPbBr_3_@MOF-5	Ship-in-Bottle (Sequential Deposition)	MOF-5	1.28	MAPbBr_3_	-	533 (428)	38	Temperature Sensing, Metal Ion Detection [120]
CsPbBr_3_@Eu-BTC	Ship-in-Bottle (in situ Deposition)	Eu-BTC	-	CsPbBr_3_	-	528 (593,618)	-	Temperature Sensing [121]
MAPbX_3_@Pb-MOF	Ship-in-Bottle (Direct Conversion)	Pb-MOF (Pb_2_(1,3,5-HBTC)_2_-(H_2_O)_4_)	-	MAPbCl_3_ MAPbCl_2_Br_1_ MAPbClBr_2_ MAPbBr_3_ MAPbBr_2_I MAPbBrI_2_ MAPbI_3_	- - - 10–20 - - -	406 443 487 527 582 687 746	- - - 40 - - -	Information Security [122]
MAPbBr_3_@Eu-BTC	Ship-in-Bottle (Sequential Deposition)	Eu-BTC	-	MAPbBr_3_	-	513 (593,617)	42	Information Security [123]
MAPbBr_3_@UiO-66	Ship-in-Bottle (Sequential Deposition)	UiO-66	0.69	MAPbBr_3_	-	505	-	Information Security [124]
CsPbX_3_@AMOF-1	Ship-in-Bottle (Sequential Deposition) ^d^	AMOF-1 (L = 5,5′-(1,4-phenylenebis(methylene))bis(oxy)diisophthalate)	-	CsPbCl_3_ CsPbBr_3_ CsPbI_3_	2–9 2–9 5–8	412 515 695	2 14 5	Information Security [125]
CsPbX_3_@ZJU-28	Ship-in-Bottle (Sequential Deposition)	ZJU-28	- - 23.56 - - -	CsPbCl_3_ CsPbCl_1.5_Br_1.5_ CsPbBr_3_ CsPbBr_1.5_I_1.5_ CsPbBr_0.6_I_2.4_ CsPbI_3_	7.92 9.21 11.85 12.87 13.74 15.65	- - 518 (445,478) - -	- - 62 - - -	Information Security, Temperature Sensing, Light-Emitting Diodes [126]
CsPbBr_3_@ZIF-8 CsPbBr_3_@ZIF-67	Bottle-Around-Ship	ZIF-8 ZIF-67	- -	CsPbBr_3_	5	524	-	Photocatalysis [127]
APbBr_3_@ZIF-8	Ship-in-Bottle (in situ Deposition)	ZIF-8	1.16	MAPbBr_3_ MA_0.75_EA_0.25_PbBr_3_ MA_0.5_EA_0.5_PbBr_3_ MA_0.25_EA_0.75_PbBr_3_ EAPbBr_3_	6–8 - - - -	527 493 481 455 < 440	80 - - - -	Photocatalysis [128]
CsPbBr_3_@UiO-66(NH_2_)	Ship-in-Bottle (Physical Mixing)	UiO-66(NH_2_)	-	CsPbBr_3_	10	-	-	Photocatalysis [129]
MAPbI_3_@PCN-221(Fe_x_)	Ship-in-Bottle (Sequential Deposition)	PCN-221(Fe_x_) (x = 0–1)	2	MAPbI_3_	1.8	610	-	Photocatalysis [130]
MAPbBr_3_@ MA-M(HCOO)_3_	Ship-in-Bottle (Sequential Deposition) ^d^	MA-M(HCOO)_3_ [M = Mn, Co]	-	MAPbBr_3_	5–10	520	<3	Photo-Electrochemical Activity [131]
APbBr_3_@Cr-MIL-101	Ship-in-Bottle (Sequential Deposition)	Cr-MIL-101 [(Cr_3_O(OH)(H_2_O)_2_(terephthalate)_3_]	2.9 3.4	CsPbBr_3_ MAPbBr_3_ FAPbBr_3_	3	440 446 450	<5	- [132]

[a] Pore size of the MOF before encapsulation. [b] Emission wavelength of MHP in the composite. The wavelength in brackets refers to that of the MOF host. [c] Photoluminescence quantum yield of MHP in the photoactive porous composite. [d] Synthesis of photoactive porous composite carried out in the absence of solvent. [e] Grain size of perovskite film.

## Data Availability

Not applicable.
